# Reorganization of 3D chromatin architecture in doxorubicin-resistant breast cancer cells

**DOI:** 10.3389/fcell.2022.974750

**Published:** 2022-08-05

**Authors:** Xuelong Wang, Jizhou Yan, Zhao Ye, Zhiqiang Zhang, Sheng Wang, Shuang Hao, Baiyong Shen, Gang Wei

**Affiliations:** ^1^ Department of General Surgery, Pancreatic Disease Center, Ruijin Hospital, Shanghai Jiaotong University School of Medicine, Shanghai, China; ^2^ CAS Key Laboratory of Computational Biology, Shanghai Institute of Nutrition and Health, University of Chinese Academy of Sciences, Chinese Academy of Sciences, Shanghai, China; ^3^ Research Institute of Pancreatic Diseases, Shanghai Jiaotong University School of Medicine, Shanghai, China; ^4^ Department of Developmental Biology, Institute for Marine Biosystem and Neurosciences, Shanghai Ocean University, Shanghai, China; ^5^ Department of Endocrinology and Metabolism, Huashan Hospital, Shanghai Medical College, Fudan University, Shanghai, China; ^6^ Institute of Translational Medicine, Shanghai Jiaotong University, Shanghai, China; ^7^ Key Laboratory of Breast Cancer in Shanghai, Department of Breast Surgery, Fudan University Shanghai Cancer Center, Shanghai, China

**Keywords:** 3D genome reorganization, doxorubicin resistance, breast cancer, compartment switching, TAD boundary, enhancer-promoter interaction

## Abstract

**Background**: Doxorubicin resistance remains a major therapeutic challenge leading to poor survival prognosis and treatment failure in breast cancer. Although doxorubicin induces massive changes in the transcriptional landscape are well known, potential diagnostic or therapeutic targets associated with the reorganization of three-dimensional (3D) chromatin architecture have not yet been systematically investigated.

**Methods:** Here we performed *in situ* high-throughput chromosome conformation capture (Hi-C) on parental and doxorubicin-resistant MCF7 (MCF7-DR) human breast cancer cells, followed by integrative analysis of HiC, ATAC-seq, RNA-seq and TCGA data.

**Results:** It revealed that A/B compartment switching was positively correlated to genome-wide differential gene expression. The genome of MCF7-DR cells was spatially reorganized into smaller topologically associating domains (TADs) and chromatin loops. We also revealed the contribution of increased chromatin accessibility and potential transcription factor families, including CTCF, AP-1 and bHLH, to gained TADs or loops. Intriguingly, we observed two condensed genomic regions (∼20 kb) with decreased chromatin accessibility flanking TAD boundaries, which might play a critical role in the formation or maintenance of TADs. Finally, combining data from TCGA, we identified a number of gained and lost enhancer-promoter interactions and their corresponding differentially expressed genes involved in chromatin organization and breast cancer signaling pathways, including *FA2H, FOXA1* and *JRKL*, which might serve as potential treatment targets for breast cancer.

**Conclusion:** These data uncovered a close connection between 3D genome reorganization, chromatin accessibility as well as gene transcription and provide novel insights into the epigenomic mechanisms involving doxorubicin resistance in breast cancer.

## Introduction

Breast cancer is one of the cancers with the highest morbidity and mortality rates, and its incidence has progressively increased over the last few decades in the female population (Sung et al., 2021). Although significant progress in basic and translational breast cancer research has been made in recent years, drug resistance in cancer cells remains a major obstacle to effective chemotherapy (Hammond et al., 2016). For breast cancer treatment, doxorubicin, a cytotoxic anthracycline antibiotic, is one of the most effective chemotherapeutic drugs. However, resistance to doxorubicin is an almost inevitable consequence, especially in metastatic breast cancer, leading to poor patient prognosis and survival for the majority of patients.

Doxorubicin, also known as adriamycin, is an anticancer drug effectively used in a variety of cancers, being first isolated from *S. peucetius var. caesius* (Arcamone et al., 1969). The chemical structure of doxorubicin comprises a tetracyclic ring with two of the groups being adjacent quinone-hydroquinones and a sugar, daunosamine, attached to ring A with a glycosidic bond (Meredith and Dass, 2016). Two main mechanisms of action have been proposed to explain the anticancer effects of doxorubicin: the first one is intercalation of DNA and inhibition of topoisomerase II, and the second one is generation of reactive oxygen species (ROS) (Thorn et al., 2011; Meredith and Dass, 2016). The molecular mechanisms of doxorubicin action in either cell death or cell growth arrest through various cellular processes, such as autophagy, senescence and apoptosis (Nitiss, 2009; Thorn et al., 2011; Meredith and Dass, 2016). Despite various genes, non-coding RNAs, signaling pathways, and chromatin accessibility changes have been reported to be involved in resistance to doxorubicin (Tormo et al., 2019; Ciocan-Cartita et al., 2020; Tanaka et al., 2020; Wang et al., 2021), little is known that the reorganization of 3D genome architecture in doxorubicin-resistant breast cancer cells.

The recently developed *in situ* Hi-C technique enables detection and visualization of genome-wide all-by-all chromatin interactions within a 3D space (Lieberman-Aiden et al., 2009; Lafontaine et al., 2021), which exhibit spatiotemporal variability at multiple scales including whole chromosomes, A/B compartments, topologically associating domains (TADs) and chromatin loops (Dixon et al., 2012; Rao et al., 2014). Increasing evidence suggests that 3D genome reorganization plays a critical role in development, cell differentiation and diseases (Lupianez et al., 2015; Wu et al., 2017; Zheng and Xie, 2019; Chen et al., 2020). It has been reported that A/B compartment switching is closely related to changes in gene expression not only between normal breast epithelial cells and breast cancer cells (Barutcu et al., 2015) but also between parental and endocrine-resistant breast cancer cells (Zhou et al., 2019). The size of the TADs is significantly smaller in prostate cancer relative to normal prostate epithelial cells (Taberlay et al., 2016). Disruptions of TADs lead to pathogenic rewiring of gene-enhancer interactions (Lupianez et al., 2015).

In the present study, we applied integrated analyses combining *in situ* Hi-C data, assay for transposase-accessible chromatin with high-throughput sequencing (ATAC-seq) data, and mRNA sequencing data on the parental and doxorubicin-resistant MCF7 cell lines, and systematically investigated global alterations in the 3D chromatin architecture associated with differential gene transcription, including compartments, TADs, and enhancer-promoter interactions.

## Materials and methods

### Cell lines and cell culture

The MCF7 and doxorubicin-resistant MCF7 cell lines were originally obtained from the Cell Bank of the Type Culture Collection of the Chinese Academy of Sciences (Shanghai, China) and cultured as previously described (Wang et al., 2021). Briefly, the cells were maintained in monolayer cultures in Dulbecco’s Modified Eagle’s medium (KEL Biotech, China) supplemented with 2 mM L-Glutamine (Gibco, USA), 100 mM sodium pyruvate (Gibco, USA), 10% FBS (KEL Biotech, China), 50 units/mL penicillin and 50 μg/ml streptomycin (Sigma-Aldrich, USA). Both MCF7 and MCF7-DR cells were grown in a humidified atmosphere of 5% CO_2_ at 37°C in 10-cm dishes. Thereafter, the medium was renewed every 2–3 days until ready for sub-culturing. At about 80% confluence, cells were serially passaged by 1:4 dilution after trypsinization. The resistant-derived cells were maintained in 1 μM doxorubicin hydrochloride (MedChem Express, USA), and the parental MCF7 cells were grown in a drug-free medium for comparison. The MCF7-DR cells were maintained in the doxorubicin-free medium for about one week prior to further experiments.

### Hi-C library generation and sequencing

The preparation of *in situ* Hi-C libraries was optimized according to previous protocols (Rao et al., 2014; Placek et al., 2017). Three million cells were fixed with freshly made 1% formaldehyde (in PBS) for 10 min at room temperature for each sample. The reaction was quenched by adding 2 M glycine to a final concentration of 0.2 M for 5 min at room temperature. Fixed cells were lysed in lysis buffer (10 mM Tris-HCl pH 8.0, 10 mM NaCl and 0.2% IGEPAL CA-630 with proteinase inhibitor) for 1 h at 4°C with rotation. The supernatant was discarded and the pellet was gently resuspended in 1 × CutSmart Buffer (NEB, B7204) containing 0.1% SDS and incubated at 65°C for 10 min. Permeabilization was quenched by adding 10% Triton X-100 and incubated at 37°C for 1 h. Chromatin was digested overnight at 37°C with rotation by adding the DpnII restriction enzyme (NEB, R0543M). Digested chromatin ends were filled and marked with biotin-14-dATP (Thermo Fisher, 19524016) using a large Klenow fragment of DNA polymerase I (NEB, M0210) for 1 h at 37°C. Samples were then diluted and ligated using T4 DNA ligase (Thermo Fisher, EL0013) at 16°C overnight. Chromatin was de-cross-linked, and proteins were removed by proteinase K, followed by phenol/chloroform extraction coupled with isopropanol precipitation using glycogen as a carrier. Biotin-14-dATP from unligated DNA ends was removed by T4 DNA polymerase (NEB, M0203) at 12°C for 2 h. DNA was purified by phenol/chloroform and sonicated using a Covaris S220 (Covaris, USA) to approximately 300–600 bp. Biotinylated DNA fragments were then pulled down using Dynabeads MyOne Streptavidin C1 beads (Thermo Fisher, 65001). The DNA ends were subsequently repaired and phosphorylated using T4 DNA polymerase (NEB, M0203), Klenow fragment of DNA polymerase I (NEB, M0210), and T4 Polynucleotide Kinase NK (NEB, M0201). The DNA was further prepared for sequencing using the VAHTS Universal DNA Library Prep Kit for Illumina V3 (Vazyme Biotech, ND607) and barcoded adaptors were ligated using TruePrep Index Kit V2 for Illumina (Vazyme Biotech, TD202). Each Hi-C library was indexed uniquely and amplified by 12 cycles of PCR for all samples. PCR purification and size selection was carried out using the VAHTS DNA Clean Beads (Vazyme Biotech, N411). Library quantity was assessed using a Qubit 3.0 Fluorometer (Invitrogen, USA). For each individual, the library was sequenced with 150 bp paired-end reads on the Illumina HiSeq X Ten platform (Novogene Biotech, China).

### Hi-C data processing

The raw sequenced Hi-C reads were first mapped to the human genome hg19 with BWA using the BWA-MEM algorithm (Li and Durbin, 2010). The read pairs which were not uniquely mapped, dangling end reads, self-circles and same-fragment reads, and other invalid Hi-C reads were discarded. Details about data quality are summarized in Supplementary Table S1 and Supplementary Figure S1. The processing of valid mapped Hi-C reads was carried out using the HiCExplorer suite of tools (v3.6) according to the documentation provided with the software (Ramirez et al., 2018). After removing duplication, reads were used to generate raw Hi-C matrices in.h5 format at 5 kb, 20 kb, 200 kb, and 1 Mb resolution using hicBuildMatrix for each sample. The hicQC was used to generate summary tables and plots of quality control (QC) measures for all samples. Correlation analysis and generation of the heatmap and scatter plots were performed by hicCorrelate to validate the reproducibility of the Hi-C data.

### Heatmap of chromatin interaction frequency and visualization

Hi-C matrices of replicates were merged using hicSumMatrices, and then normalized and corrected (Imakaev et al., 2012) afterward using hicNormalize and hicCorrectMatrix, respectively. The application hicPlotMatrix was used to generate the heatmaps to visualize interaction frequencies with 5- or 10-kb resolution for local views, a 200-kb resolution for a whole-chromosome view, and a 1-Mb resolution for a whole-genome view. To analyze differential interaction frequencies between samples, the application hicCompareMatrices was used to generate log 2 ratios of interaction frequency matrices between MCF7 and MCF7-DR datasets. We also converted valid read pairs into.cool and other format files with hicConvertFormat command for downstream analysis and visualization.

### Analyses of A and B compartments

To investigate sub-nuclear compartments, the makeTagDirectory supplied in HOMER (v4.10) (Heinz et al., 2010) was used to create tag directories with the option “-tbp 1” to remove duplicate read pairs. In order to remove paired-end reads likely representing continuous genomic fragments or re-ligation events, self-ligations, and reads originating from regions with unusually high tag density, makeTagDirectory was used with the following options: removePEbg, -removeSelfLigation, -removeSpikes 10000 5. Then valid contact read pairs of samples were applied to obtain the first principal component (PC1) values corresponding to type A/B (active/inactive) compartments using the analyzeHiC and runHiCpca.pl scripts in HOMER (v4.10) at 100-kb resolution with parameter “-res 100000”. The switching compartments (“A to B” or “B to A”) are defined as genomic regions in which one type of compartmentalization is observed in one cell line and the other compartment type in the second cell line.

### Identification of TADs, long-range interactions and chromatin loops

TADs and their corresponding boundaries were identified at different resolution with hicFindTADs command in HiCExplorer suite (v3.6) (Ramirez et al., 2018; Wolff et al., 2020) with parameters “--minDepth 15000 --maxDepth 75000 --step 7500 --delta 0.01 --thresholdComparisons 0.01 --correctForMultipleTesting fdr”. TAD-separation scores, or the so-called TAD insulation score, were calculated to measure the degree of separation between the left and right regions at each Hi-C matrix bin, and then TADs were called as those positions having a local TAD-separation score minimum (Ramirez et al., 2018).

After chromatin compartments analysis with HOMER suites (v4.10) (Heinz et al., 2010), the analyzeHiC command was used to obtain long-range intra-chromosomal contacts (>20 kb) at 5-kb resolution with options: res 5000 -zscore 1 -interactions -pvalue 0.05 -minDist 20000 -maxDist 1000000.

Chromatin loops were identified based on a strict candidate selection, negative binomial distributions and Wilcoxon rank-sum tests as described in the previous publication (Rao et al., 2014) using hicDetectLoops (Ramirez et al., 2018) at 5-kb resolution with parameters: -maxLoopDistance 1000000 --pValue 0.05.

### Classification of TAD boundaries and loop anchors

By comparing the identified TAD boundaries and their separation scores, it was defined as a gained TAD boundary that was only identified as a boundary in MCF7-DR cells and possessed a lower TAD-separation score (i.e., higher insulation strength) compared to the parental cells; a lost TAD boundary was associated with a higher TAD-separation score (i.e., lower insulation strength) in MCF7-DR cells and was only detected in MCF7 cells; the boundaries detected in both MCF7 and MCF7-DR cells were defined as stable TAD boundaries.

According to the genomic locations, the chromatin loops were classified into stable, gained or lost: stable, loops present in both MCF7 and MCF7-DR cells; gained, loops present in the MCF7-DR cells but not in the MCF7 cells; lost, loops present in the loops but not in the MCF7-DR cells.

### Calculation of aggregate peak analysis score

To measure the aggregate strength of stable, lost or gained chromatin loops, APA score was calculated as described previously (Rao et al., 2014) and visualized by plotting a cumulative stack of sub-matrices around detected loop coordinates at 5-kb resolution using the apa-analysis script in HiCPeaks software (Salameh et al., 2020) with default parameters.

### Analysis of transcription factor motif enrichment and occurrence probability

To identify transcription factors potentially involved in the formation of TAD boundaries or loop anchors, motif enrichment analysis was performed using the findMotifsGenome.pl script from HOMER suite (v4.10) (Heinz et al., 2010) with the parameter -size -2500,2500. Only TF motifs with a *p* value lower than 0.01 were kept for downstream analysis. Then the annotatePeaks.pl (Heinz et al., 2010) was used to calculate the occurrence probability of a certain TF flanking the center of TAD boundaries or loop anchors with the parameter -size 20000, and results were visualized with the ggplot2 R package (https://ggplot2.tidyverse.org).

### Identification of differential enhancer-promoter interactions

The “bedtools intersect” command of BEDTools (v2.30.0) (Quinlan, 2014) was used to calculate overlap regions between loop anchor (5-kb resolution), transcription start site (TSS) and enhancer. Differential enhancer-promoter interactions (EPIs) are defined as Hi-C-derived chromatin loops whose anchors are overlapped with enhancers or TSSs, respectively, requiring a minimum 1-bp overlap. The consensus enhancers of the MCF7 cell line are downloaded from EnhancerAtlas 2.0 (Gao and Qian, 2020), which were predicted based on multiple high throughput experimental datasets. Taking into account the impact of sequencing depth and resolution of Hi-C data, the exact position of hg19 TSS for each gene was used for EPIs analysis.

### Integrated analysis of ATAC-seq and RNA-seq data

ATAC-seq and RNA-seq data of MCF7 and MCF7-DR cells were obtained from our previous publication and are available in the NCBI Gene Expression Omnibus (GEO) repository under the accession number GSE174152 (Wang et al., 2021), including differentially expressed genes, hyper- and hypo-accessible regions, and normalized coverage (BigWig) files used in this study. The heatmaps and average profiles of mRNA-seq and ATAC-seq signals around TAD borders or loop anchors were generated using the plotHeatmap and plotProfile scripts in deepTools (v3.5.1) (Ramirez et al., 2014).

### Enrichment analysis of GO terms and KEGG pathways

Functional annotation enrichment analysis with GO terms and KEGG pathways were performed using the clusterProfiler R package (Yu et al., 2012) for gene sets of interest. The enriched terms and pathways were considered to be significant with *p* value < 0.05.

### TCGA-BRCA data analysis

Based on the clinical and genomic data from The Cancer Genome Atlas (TCGA) Breast Invasive Carcinoma (BRCA), the breast cancer samples were divided into two subgroups based on whether the doxorubicin (a.k.a. adriamycin) was used alone, namely the doxorubicin alone group (Dox^+^; *n* = 363) and the other group (Dox^−^; *n* = 762). The mRNA expression levels (FPKM) of representative genes were compared between the Dox^+^ and Dox^−^ subgroups using the Wilcoxon rank-sum test in R software (v 4.1.2). Pearson’s correlation coefficient was then calculated to assessing the associations between critical DEGs using the “corrplot” R package. The Kaplan-Meier survival curves and log-rank test were performed by utilizing the “survival” and “survminer” packages in R to analyze survival differences. *p* < 0.05 were regarded as statistically significant.

### Statistical analysis and data visualization

If not specified, all bioinformatics analyses were performed with R software (v4.1.2) to compute statistics and generate plots throughout this manuscript. Wilcoxon rank-sum test or Student’s t-test was used to assess significance in paired comparisons. Pearson correlation coefficient was used to compute correlations between two conditions. Fold change, *p* values and false discovery rate (FDR) were calculated in the analysis. Significant differences for all quantitative data were considered when *p < 0.05, **p < 0.01, ***p < 0.001, and ****p < 0.0001.

## Results

### Compartment switching is positively correlated with genome-wide differential gene expression associated with doxorubicin resistance

**FIGURE 1:**
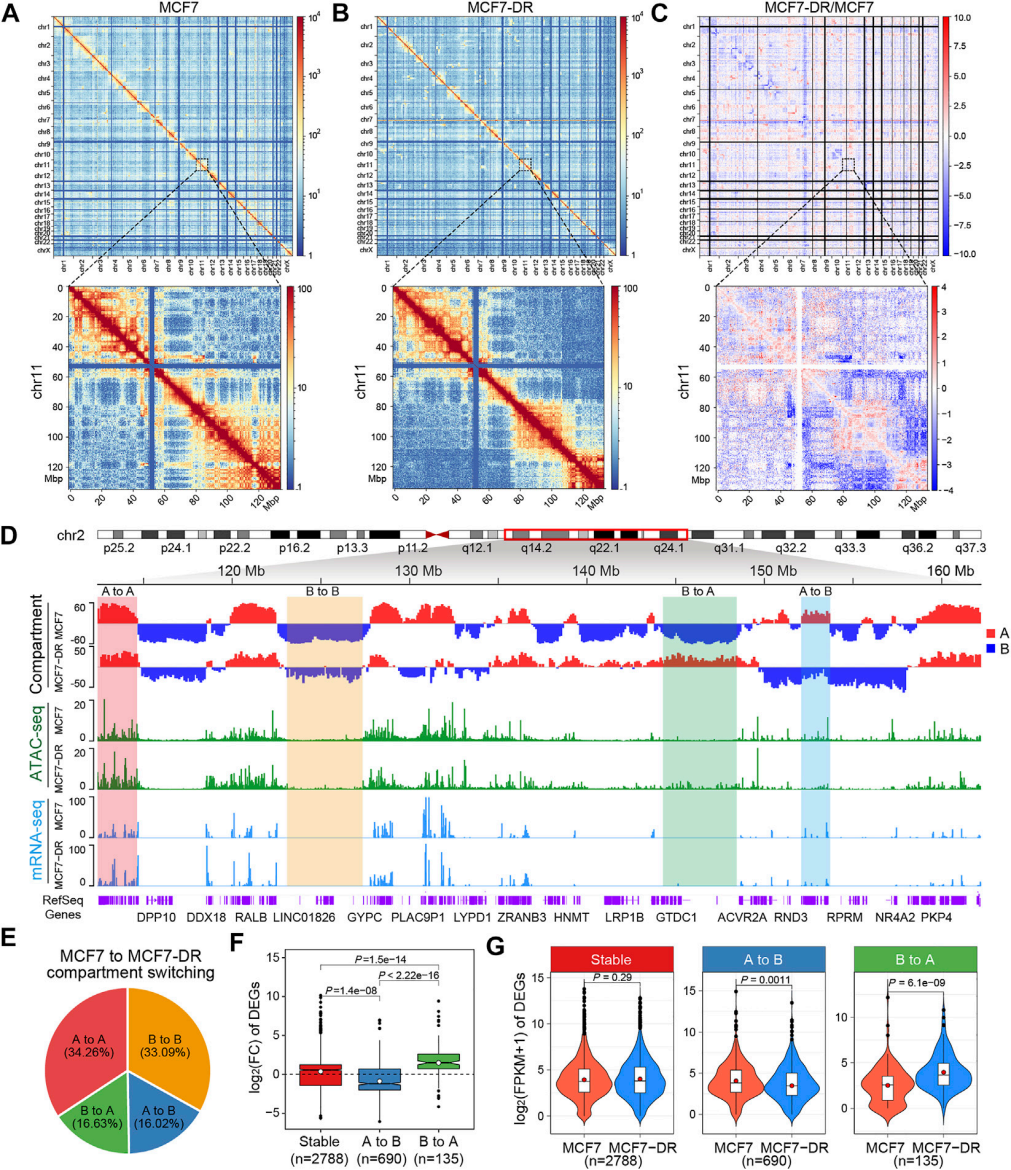
Compartment switching is associated with differential gene transcription. **(A,B)** Genome-wide all-by-all Hi-C interaction heatmaps at 1-Mb resolution in MCF7 **(A)** and MCF7-DR **(B)** cells. The chromosomes are stacked in numerical order from left to right and top to bottom. The color bar depicts the frequency of Hi-C interactions. The lower panels are enlargements of the chr11 at 200-kb resolution in the upper panels. **(C)** Genome-wide heatmap showing the log2 ratio comparisons of Hi-C interaction frequencies at 1-Mb resolution in MCF7-DR cells compared to parental MCF7 cells. The log2 ratio values are shown according to the color scale. The lower panel represents the enlargement of the chr11 at 200-kb resolution in the upper panel. **(D)** A representative image from the Integrated Genome Browser (IGV) that shows the open A-type (red) and closed B-type (blue) compartmentalization of chr2 at 100-kb resolution. Examples of regions with either stable or differential compartmentalization are shaded in different colors. Tracks showing ATAC-seq and RNA-seq read coverage are indicated by green and blue bars, respectively. **(E)** The genomic compartment changes at 100-kb resolution between MCF7 and MCF7-DR genomes. “A” and “B” represent the open and closed compartments, respectively. “A to A” denotes compartments that are open in both cell lines, “B to B” represents compartments that are closed in both cell lines, “A to B” denotes compartments that are open in MCF7 but closed in MCF7-DR, and “B to A” denotes compartments that are closed in MCF7 and open in MCF7-DR. **(F)** Box plot showing MCF7-DR/MCF7 log2 fold change (FC) of DEGs residing at regions for different compartmental switch categories (100-kb resolution). Statistical significance was determined by the Wilcoxon rank-sum test. The black horizontal dotted line represents the log_2_(FC) = 0. In each box plot, the median and mean values are indicated by the horizontal black line and the white dot, respectively. The number of DEGs associated with compartment switching was marked below each box. **(G)** Violin plots showing expression level [log_2_(FPKM+1)] of DEGs residing at regions for different compartmental switch categories. The horizontal black line within each white box indicates the median value while the red dot represents the mean value. For comparisons between MCF7 and MCF7-DR cell lines, statistical significance was calculated using the Wilcoxon rank-sum test.

To probe the reorganization of 3D chromatin architecture in doxorubicin-resistant breast cancer, we generated Hi-C libraries from two independent biological replicates for doxorubicin-resistant MCF7 (MCF7-DR) cells and parental MCF7 cells (Figures 1A,B; Supplementary Figure S1), which are human breast cancer cells derived from malignant breast adenocarcinoma tissue. After sequence quality filtering, about 501 and 488 million valid contacts were obtained from the MCF7 and MCF7-DR combined replicate Hi-C libraries, respectively (Supplementary Figures S1A–D and Supplementary Table S1), with high reproducibility between the biological replicates (Supplementary Figure S2). Then the normalized Hi-C contact matrices were visualized as chromosome versus chromosome heatmaps, where darker red colors represent higher interaction frequency, while darker blue colors indicate lower interaction frequency (Figures 1A,B and Supplementary Figures S1E,F). It revealed that intra-chromosomal interactions were much more frequent than inter-chromosomal interactions (Figures 1A,B and Supplementary Figures S1E,F), reflecting “chromosome territories” as previously observed by Hi-C data and cell imaging (Lieberman-Aiden et al., 2009; Cremer and Cremer, 2010).

Increasing evidence suggests that the mammalian genome is spatially organized into two major types of compartments, “A” and “B”: A compartments are mainly composed of euchromatin which is transcriptionally active whereas B compartments are characterized by inactive heterochromatin and exhibit high chromatin density (Dixon et al., 2012; Dekker and Mirny, 2016; Chen et al., 2020). To address the correlation between A/B compartment switching and differential gene expression in MCF7-DR cells, we determined the compartment types of the genome at 100-kb resolution. Genome-wide comparison of interaction matrices between MCF7 and MCF7-DR cells uncovered varying degrees of changes in chromosomal interactions and compartmentalization (Figures 1C,D). The majority of the compartmentalization was similar in both MCF7 and MCF7-DR cells, with 34.26% of the genome consisting of constitutive A compartments and 33.09% consisting of constitutive B compartments (Figures 1D,E). A total of 16.02% of genomic regions switched from A compartments in MCF7 cells to B compartments in MCF7-DR cells and 16.63% of genomic regions showed the opposite alteration from B compartments in MCF7 cells to A compartments in MCF7-DR cells (Figures 1D,E).

To further understand the link between compartment switching and differential gene expression associated with doxorubicin resistance, in combination with mRNA-seq data (Wang et al., 2021), we identified 2788, 690 and 135 differentially expressed genes (DEGs) that were located within stable (“A to A” or “B to B”), “A to B” and “B to A” compartment regions, respectively (Figure 1F). It showed that most of the DEGs located within genomic regions converted from B to A compartment were significantly up-regulated with a higher log 2 fold change, while the DEGs located in regions with a compartment switch from A to B were mostly repressed (Figure 1F). Moreover, we found that the differential gene expression within the switching compartments was positively correlated with the direction of the switch: The expression level of DEGs located in genomic regions converted from the B to the A compartment in MCF7-DR cells was significantly higher than those that in MCF7 cells, and vice versa (Figure 1G). These results suggested a positive relationship between genome-wide compartment switching and differential expression of their corresponding genes.

### TADs and chromatin loops are on average smaller in MCF7-DR cells

**FIGURE 2:**
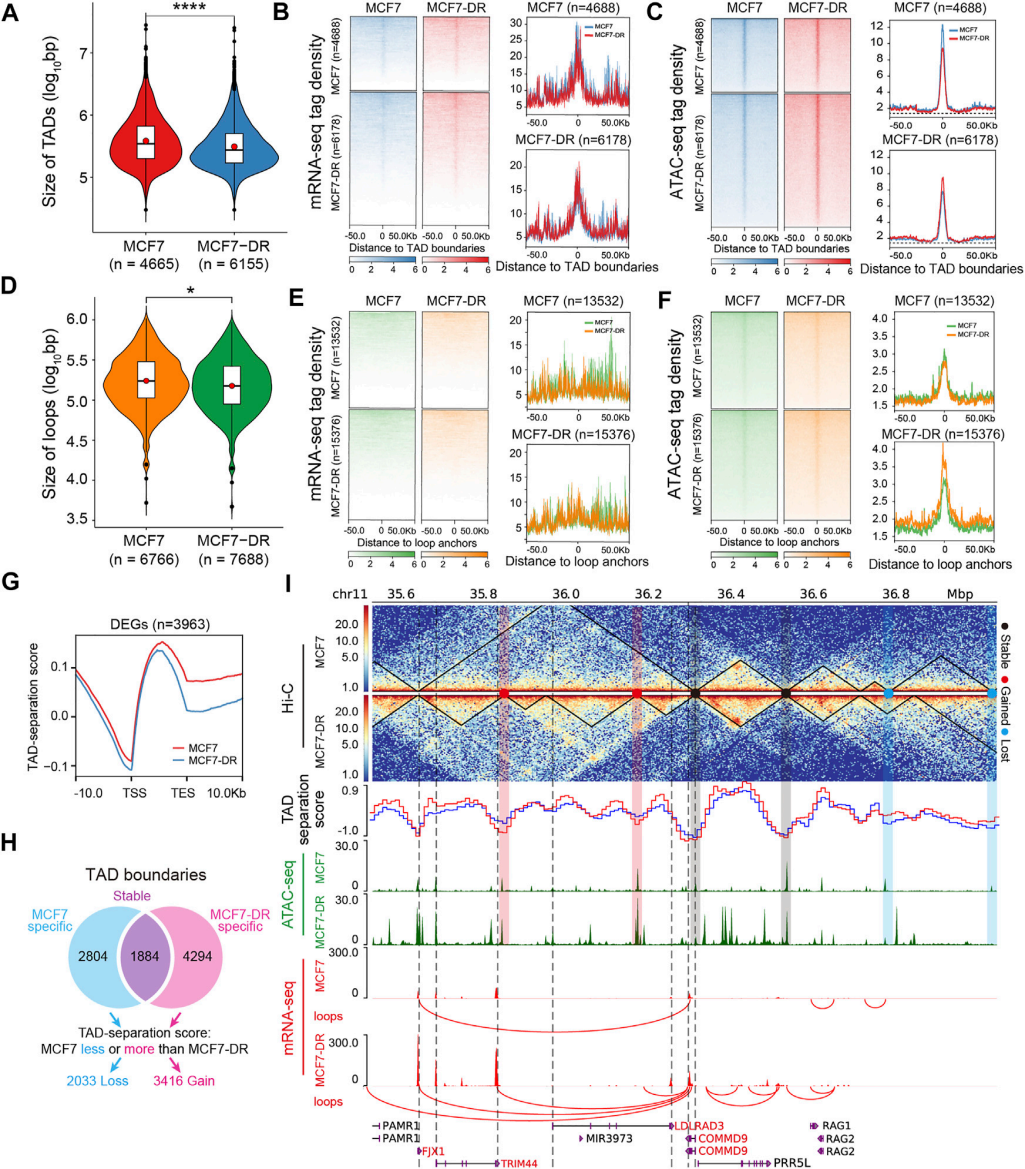
Characteristics and genome-wide changes in TADs and loops. **(A)** Violin plot showing the size distribution of TADs at 5-kb resolution for whole-genome. The violin plot below shows the number of identified TADs in MCF7 (*n* = 4665) and MCF7-DR (*n* = 6155) cells. *p* values were calculated by Wilcoxon rank-sum test in R software, ****p < 0.0001. **(B,C)** Heatmaps and average profiles displaying normalized tag density (RPKM) of mRNA-seq **(B)** and ATAC-seq **(C)** data over the identified TAD boundaries across a genomic window -50 kb upstream and +50 kb downstream from the center of TAD boundaries in MCF7 and MCF7-DR cells. The top panels show read signals over the 4688 TAD boundaries identified in MCF7 cells and the bottom panels show read signals over the 6178 TAD boundaries identified in MCF7-DR cells. **(D)** Violin plot showing the size distribution of chromatin loops at 5-kb resolution for the whole genome. The number of identified loops in MCF7 (*n* = 6766) and MCF7-DR (*n* = 7688) cells is displayed below the violin plot. *p* values were calculated by Wilcoxon rank-sum test in R software, *p < 0.05. **(E,F)** Heatmaps and average profiles displaying normalized tag density (RPKM) of mRNA-seq **(E)** and ATAC-seq **(F)** data located within 50 kb upstream and downstream of the center of loop anchors in MCF7 and MCF7-DR cells. The top panels show read signals over the 13532 loop anchors identified in MCF7 cells and the bottom panels show read signals over the 15376 loop anchors identified in MCF7-DR cells. **(G)** A profile of the average TAD-separation scores across the gene body and the 10 kb upstream of TSS and 10 kb downstream of TES of all DEGs. **(H)** A schematic flow diagram showing the identification of gained, lost or stable TAD boundaries. The gained TAD boundaries are MCF7-DR specific and possess lower TAD-separation scores compared to the parental cells. The lost TAD boundaries were associated with a higher TAD-separation score (i.e., lower insulation strength) in MCF7-DR cells and were only detected in MCF7 cells; the boundaries detected in both MCF7 and MCF7-DR cells were defined as stable TAD boundaries. **(I)** A representative snapshot of the Hi-C interaction matrix at 5-kb resolution (top panel), and black lines within the heatmap demarcates the identified TADs. The black dot represents the stable TAD boundaries that are present in both cell lines, while the red and blue dots denote the gained and lost TAD boundaries in MCF7-DR cells, respectively. TAD-separation scores of MCF7 (solid blue line) and MCF7-DR (solid red line) cells were calculated to identify the degree of separation between the left and right regions at each Hi-C matrix bin. The following tracks show normalized ATAC-seq (green) and mRNA-seq (red) read coverage. The blue and red shaded areas indicate genomic regions flanking lost and gained TAD boundaries associated with decreased and increased chromatin accessibility in MCF7-DR cells compared to MCF7 cells respectively, while the grey shaded area show genomic regions flanking the stable TAD boundaries. The black vertical dashed lines indicate the TSS or TES of DEGs. The up-regulated genes are colored in red, and unchanged genes are shown in black.

Each chromosome is composed of many distinct megabase-sized chromatin domains, referred to variably as topologically associating domains (TADs) that interact more frequently within themselves than with neighboring regions (Dixon et al., 2012; Sexton et al., 2012; Ramirez et al., 2018). Disruption of TAD boundaries has been shown to affect the expression of nearby genes and link to diseases (Lupianez et al., 2015; Ibrahim and Mundlos, 2020). To capture the changes in TADs between MCF7 and MCF7-DR cells, we called TADs from iteratively corrected relative contact probability matrices at 5-kb resolution, and found that the genome of MCF7 cells comprised a total of 4665 TADs, whereas the genome of MCF7-DR cells is partitioned into 6155 TADs (Supplementary Figures S2A, S3A and Supplementary Table S2). Comparatively, MCF7-DR genome contains more TADs and the average TAD sizes for most chromosomes are smaller than in MCF7 cells (Figures 2A,I, Supplementary Figures S3A,E).

Chromatin loops are another level of hierarchical genome organization, ranging from Kbs to Mbs, and bring distal cis-regulatory elements such as enhancers into close physical proximity with promoters of target genes, which are vital for the regulation of gene transcription (Rao et al., 2014; Misteli, 2020). Using the HiCExplorer application hicDetectLoops (Ramirez et al., 2018), we identified a total of 6766 and 7688 chromatin loops from Hi-C interaction matrices at 5-kb resolution in MCF7 and MCF7-DR cells (Figure 2D, Supplementary Figure S3B and Supplementary Table S3), respectively. Changes in terms of the number and size of loops exhibited a similar trend to that of TADs (Figures 2D,I, Supplementary Figures S3B,E).

### TAD boundaries and loop anchors are more accessible and enriched with CTCF motif

In combination with ATAC-seq and RNA-seq data, we analyzed the distribution characteristics of chromatin accessibility, mRNA expression levels and TAD-separation scores around the TAD boundaries and loop anchors. Our data revealed that both TAD boundaries and loop anchors possessed lower TAD separation scores (Supplementary Figures S3C,D) and were strongly enriched for ATAC-seq signals (Figures 2C,F,I, Supplementary Figure S3E), indicating stronger insulation and higher chromatin openness at TAD boundaries and loop anchors compared to the surrounding regions. In addition, the TAD boundaries, but not loop anchors, were significantly enriched for RNA-seq signals (Figures 2B,E), and the transcription start site (TSS) and transcription end site (TES) of DEGs possessed lower TAD-separation scores indicating stronger insulation (Figure 2G). It may be due to the reason that chromatin loop anchor points provide long-range interactions between promoters and regulatory elements, especially distant intergenic enhancers with high accessibility (Rao et al., 2014). TAD boundaries are more likely to be openly accessible and transcriptionally active, which is consistent with previous findings (Ulianov et al., 2016; Huang et al., 2020).

Transcription factors (TFs) regulate gene expression through binding directly to cis-regulatory elements of target genes in a sequence-specific manner dependent on the consensus motif (Tsankov et al., 2015; Lambert et al., 2018). To examine potential TFs located within the TAD boundaries and loop anchors, we performed TF enrichment analysis using the findMotifsGenome.pl tool provided by the HOMER motif discovery software (v4.10) (Heinz et al., 2010), and the sequences of TAD boundaries and loop anchors were scanned for TF binding sites and motif occurrences. Our analysis revealed that CTCF and BORIS (also known as CTCFL) motifs were found to be significantly (P < 1e-80) enriched at both TAD boundaries (Supplementary Figures S4A,B) and loop anchors (Supplementary Figures S4E,F) with a higher binding probability in both MCF7 and MCF7-DR cells (Supplementary Figures S4C,D,G,H). Besides, we found that the TAD boundaries were also enriched with forkhead box (FOX) family members, such as FOXA1 and FOXK1 (Supplementary Figures S4A,B), and the HIC1 (Hypermethylated in cancer 1) motif was significantly enriched at both TAD boundaries and loop anchors, which is potentially related to genome-wide changes in chromatin accessibility induced by acute depletion of CTCF (Xu et al., 2021). These results suggest that combinations of sequence-specific DNA binding proteins were potentially involved in defining TAD boundaries and loop anchors of the genome.

### Increased chromatin accessibility at boundaries and stably condensed chromatin regions flanking boundaries are required for gained TAD boundary formation

**FIGURE 3:**
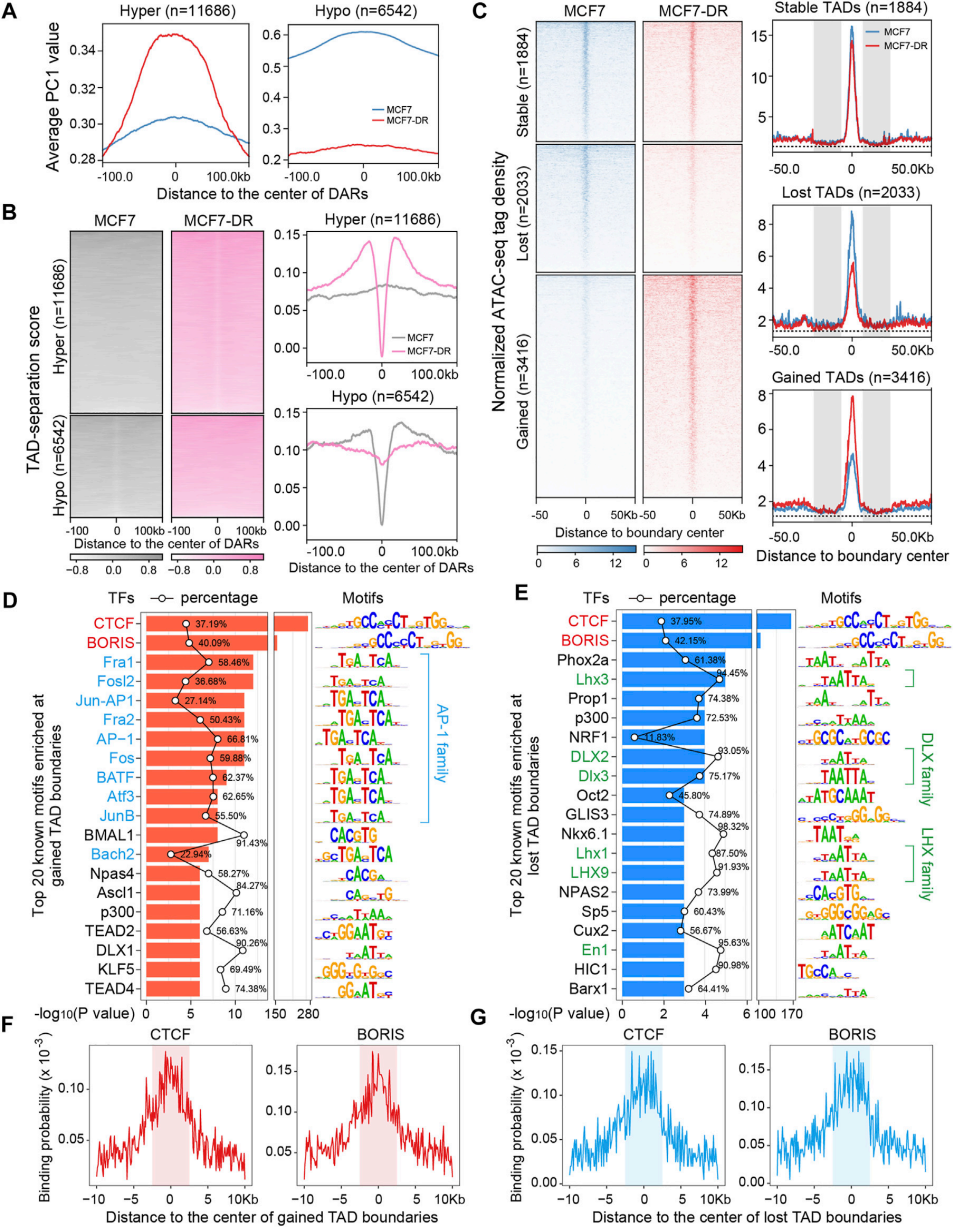
Differential chromatin accessibility and transcription factor families contribute to the formation of TAD boundaries. **(A)** Profiles depicting the distribution of average PC1 values (at 20-kb resolution) identified by the HOMER software (v4.10) surrounding the center of 11686 hyper- and 6542 hypo-accessible regions across a genomic window of ±100 kb in MCF7-DR cells compared to MCF7 cells. DARs, differentially accessible regions. **(B)** Heatmaps and average profiles of TAD-separation scores surrounding the center of 11686 hyper- (top panel) and 6542 hypo-accessible (bottom panel) regions across a genomic window of ±100 kb in MCF7-DR cells compared to MCF7 cells. **(C)** Heatmaps and profiles showing the normalized ATAC-seq tag density surrounding the center of the stable (*n* = 1884), lost (*n* = 2033) and gained (*n* = 3416) TAD boundaries across a genomic window of ±50 kb in MCF7-DR cells compared to MCF7 cells. The grey shaded areas indicate the condensed genomic regions with reduced chromatin accessibility flanking TAD boundaries. The black horizontal dashed line indicates the minimum value of normalized ATAC-seq signal around TAD boundaries. **(D,E)** Top 20 known TF motifs enriched at gained **(D)** and lost **(E)** TAD boundaries. The *p* values for motif enrichment were calculated by the HOMER software (v4.10), and a *p* value <0.01 was considered to be statistically significant by default. The percentages of target sequences with TF motifs are indicated by the black polygonal chain. The AP-1 family members are colored blue **(D)**, and the members of DLX and LHX families are colored green **(E)**. **(F,G)** The aggregate profiles show the binding probability distribution of CTCF and BORIS motifs within 10 kb around the center of gained **(F)** and lost **(F)** TAD boundaries. The shaded areas represent the regions of TAD boundaries.

Our previous work demonstrated that differentially accessible regions (DARs), including 11686 hyper- and 6542 hypo-accessible regions, were positively correlated with nearest DEGs in MCF7-DR cells (Wang et al., 2021). To further investigate the influence of chromatin accessibility changes on newly formed TADs, by comparing the TAD boundaries according to their separation scores between MCF7 and MCF7-DR cells, we classified TAD boundaries into three groups: 1884 stable, 2033 lost and 3416 gained (Figures 2H,I). It has been reported that differential chromatin accessibility is positively correlated with the alteration of A/B compartments (Hu et al., 2018; Crispatzu et al., 2021; Wang et al., 2021). Positive PC1 values corresponded to compartment A and negative PC1 values stand for compartment B. Our data showed that the average PC1 values (at 20-kb resolution) around the hyper-accessible regions (with higher chromatin accessibility) were much higher in MCF7-DR cells than in MCF7 cells, and vice versa for hypo-accessible regions (Figure 3A). Further analysis of TAD-separation scores around DARs demonstrated that the hyper-accessible regions possessed lower TAD-separation scores in MCF7-DR cells and showed a similar feature to TAD boundaries (Figure 3B, Supplementary Figure S3C). These results suggested a close association between compartmentalization, TAD boundary formation and chromatin accessibility changes. Then, we calculated the average tag density of ATAC-seq reads around the stable, lost and gained TAD boundaries and found that these gained TAD boundaries were obviously enriched for higher chromatin accessibility, and vice versa, while the stable TAD boundaries in both cell lines showed little difference (Figure 3C), demonstrating that increased chromatin accessibility is required for and contributes to the gained TAD boundary formation.

What is more, it is interesting that we have stumbled across two stably condensed chromatin regions (∼20 kb) with decreased chromatin accessibility (lower ATAC-seq signals) flanking (within ±30 kb) the stable, gained or lost TAD boundaries (Figures 2C, 3C), which have not been reported so far and maybe essentially important for the formation and/or maintenance of TAD boundaries.

### AP-1, DLX and LHX family members potentially involved in TAD reorganization

In order to identify TFs potentially involved in TAD reorganization associated with doxorubicin resistance, the sequences of changed TAD boundaries were scanned for significantly enriched TF motifs using HOMER software (v4.10) (Heinz et al., 2010). As expected, at both the gained or lost TAD boundaries, binding motifs for CTCF and BORIS were most significantly enriched (Figures 3D,E) and showed a higher binding probability (Figures 3F,G). Besides, the gained TAD boundaries were also enriched for binding motifs of AP-1 family members, including Fos (Fra1, Fra2, c-Fos), Jun (c-Jun, Junb, Jund) and Atf (Atfa, Atf2, Atf3, Batf) proteins (Figure 3D), which always bind to the DNA sequence “TGACTCA” and are commonly found in open chromatin regions enriched with ATAC-seq signals (Lambert et al., 2018; Wang et al., 2021; Zhang et al., 2022). Whereas members of DLX and LHX TF families were found to be enriched in the lost TAD boundaries (Figure 3E), which preferentially bind to a similar “TAATTA” motif (Tsankov et al., 2015; Lambert et al., 2018).

### bHLH and SOX family members potentially involved in chromatin loop changes

**FIGURE 4:**
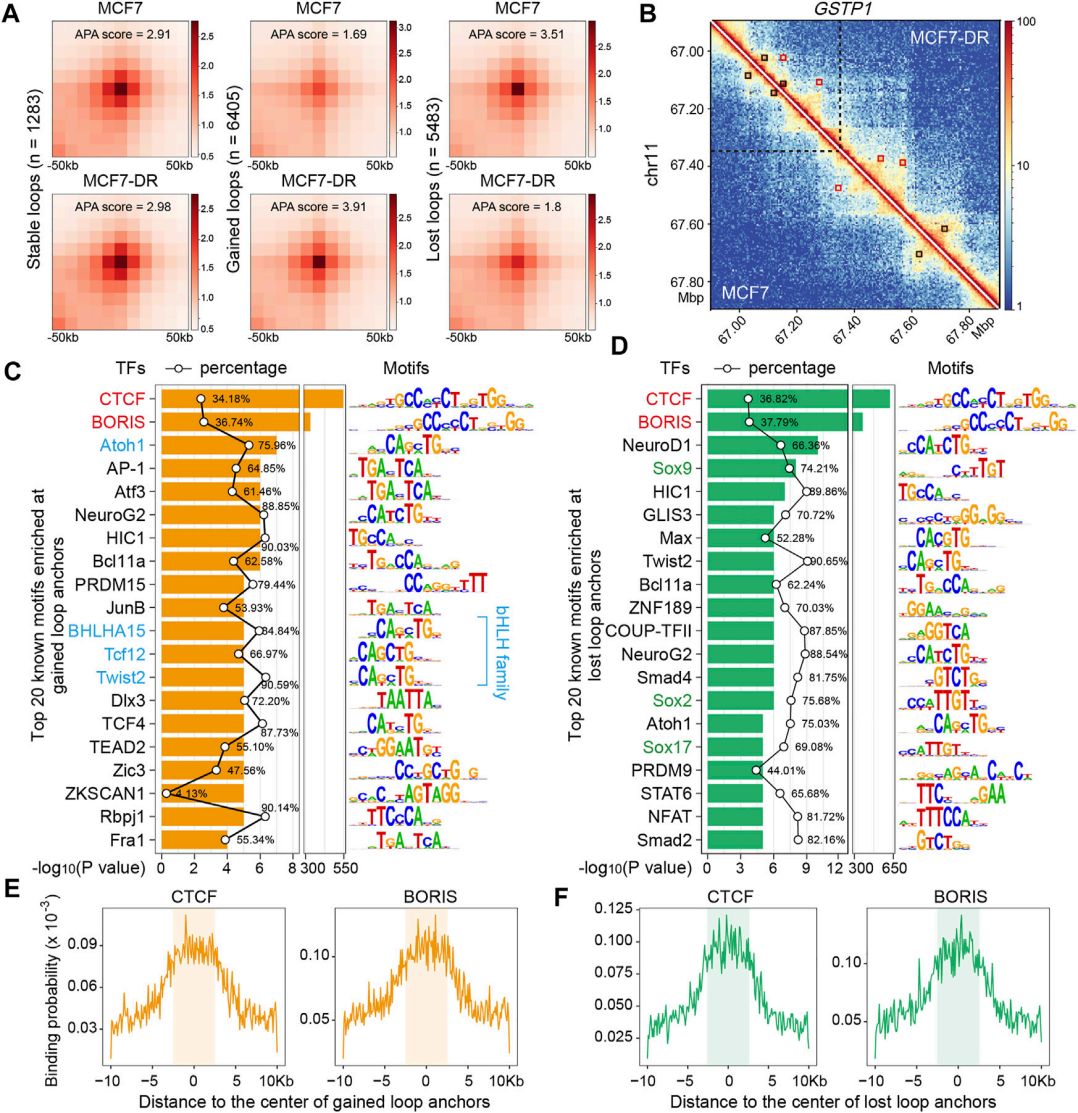
Transcription factor families potentially involved in chromatin loop changes. **(A)** Aggregate peak analysis (APA) plots measuring the aggregate strength of the stable (*n* = 1283), gained (*n* = 6405) and lost loops (*n* = 5483). APA score was calculated with HiCPeaks software using Hi-C matrix data at 5-kb resolution. **(B)** Heatmap depicting an example of differential chromatin loops (at 5-kb resolution) between MCF7 (lower left) and MCF7-DR (upper right) on chr11:66900000–67900000. The black boxes represent the stable loops that are present in both cell lines, while the red boxes denote the gained or lost loops in each cell line. The black vertical dashed lines indicate the *GSTP1* locus. **(C,D)** Top 20 known TF motifs enriched at gained **(C)** and lost **(D)** loop anchors. The *p* values for motif enrichment were calculated by the HOMER software, and a *p* value less than 0.01 is considered to be statistically significant by default. The percentages of target sequences with TF motifs are indicated by the black polygonal chain. The bHLH family members were colored blue **(C)** and the SOX family members were colored green **(D)**. **(E,F)** The aggregate profiles show the binding probability distribution of CTCF and BORIS motifs within 10 kb around the center of gained **(E)** and lost **(F)** loop anchors. The shaded areas represent the regions of loop anchors at 5-kb resolution.

To obtain potential TFs associated with chromatin loop changes, the identified loops were classified into three types based on the genomic location of loop anchors: 1283 stable loops, 6405 gained loops and 5483 lost loops (Figures 4A,B). Aggregate peak analysis (APA) was performed to assess the aggregate strength of these loops, which showed that there was a significant enrichment with a higher APA score for gained loops in MCF7-DR cells, and vice versa (Figure 4A). Then, the sequences of gained or lost loop anchors were used for enrichment analysis of known TF motifs and it revealed that, besides CTCF and BORIS, members of bHLH family, such as ATOH1, BHLHA15 and TCF12, were also significantly enriched at gained loop anchors, which preferentially bind to a similar “CAGCTG” motif, whereas the lost loop anchors were potentially enriched with SOX family members (Figures 4C,D). Although the CTCF motif was most significantly enriched and possessed a higher binding probability (Figures 4C–F), members of bHLH and SOX families were potentially involved in chromatin loop changes associated with doxorubicin resistance in MCF7 cells.

### Gained or lost enhancer-promoter interactions are positively correlated with their corresponding DEGs

**FIGURE 5:**
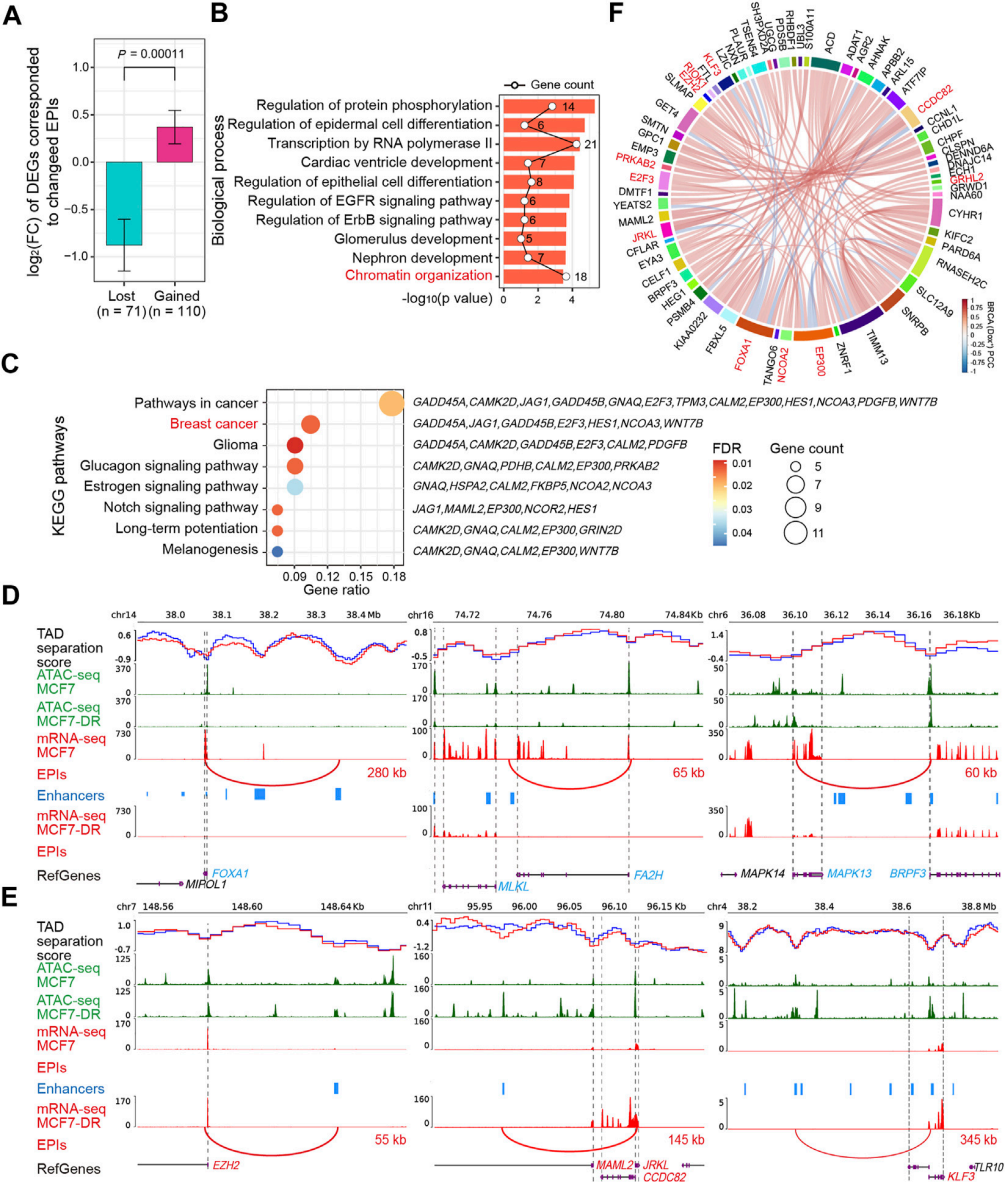
Analysis of changed enhancer-promoter interactions and their corresponding DEGs. **(A)** The average log_2_(FC) of 71 and 110 DEGs corresponded to lost and gained enhancer-promoter interactions (EPIs), respectively. Statistical significance (*p* value) was evaluated using the Wilcoxon rank-sum test in R software. The number of DEGs associated with changed EPIs is displayed below the corresponding bar. FC, fold change. **(B)** Bar plot of significantly enriched GO terms under the “biological process” category for the identified DEGs in **(A)**. The polygonal chain in black shows the gene count related to each term. **(C)** Bubble plot representing KEGG pathways significantly enriched by the identified DEGs in **(A)**. Gene ratio refers to the ratio of the number of genes enriched in the pathway to the total number of genes. **(D,E)** Representative snapshots of TAD-separation scores, ATAC-seq signals (green), mRNA-seq signals (red), enhancers (blue), changed EPIs (red) associated with down-regulated *FOXA1*, *FA2H*, *MAPK13* genes (D, blue), and up-regulated *EZH2, JRKL*, *KLF3* (E, red) genes in MCF7-DR cells compared to parental MCF7 cells. TAD-separation scores of MCF7 and MCF7-DR cells are shown as solid blue and red lines, respectively. Lost or gained EPIs are displayed as arcs between the two genomic loci and the distance of an EPI is marked next to the arc. The black vertical dashed lines indicate the TSS or TES of DEGs. **(F)** Chord diagram showing the correlation between the identified DEGs corresponded to EPIs using the normalized expression matrix from breast invasive carcinoma (BRCA) patients treated with doxorubicin (adriamycin) alone in the TCGA cohort (Dox^+^, *n* = 363). The connecting chord represents a Pearson’s correlation coefficient (PCC) > 0.5 or < −0.5. Some representative DEGs displayed in Figures 4C–E, Supplementary Figure S6 were colored in red.

Long-range chromatin interactions including enhancer-promoter interactions (EPIs) are increasingly being recognized as an important mechanism involved in epigenetic regulation of gene expression and chromatin organization (Rao et al., 2014; Dekker and Misteli, 2015; Rowley and Corces, 2018; Zheng and Xie, 2019). Using the analyzeHiC HOMRE command (Heinz et al., 2010), we identified a number of long-range chromatin interactions (FDR <0.05) spanning more than 20 kb and less than 1 Mb in both the MCF7 and MCF7-DR cells (Supplementary Figure S5), and it showed that the genomic regions around significantly up-regulated genes tend to be associated with more chromatin interactions and higher accessibility, such as *ETS1*, *EGFR* and *WTAPP1* (Supplementary Figures S5A–C), whereas the regions near down-regulated genes were associated with fewer chromatin interactions and lower accessibility, such as *DYM*, *CDYL2* and *UBR5* (Supplementary Figure S5D–F), indicating a close relationship between long-range chromatin interaction changes and DEGs. To identify lost or gained EPIs and their target DEGs, we downloaded the consensus enhancers of MCF7 cell line from EnhancerAtlas 2.0 (Gao and Qian, 2020), which were predicted based on multiple high throughput experimental datasets. We defined a differential EPI to be a gained or lost chromatin loop whose an anchor overlapped the TSS of a certain gene and the second anchor overlapped at least one distal enhancer. Finally, we identified 116 gained and 72 lost EPIs corresponding to 110 and 72 DEGs (Figure 5A and Supplementary Table S4), respectively. Remarkably, it showed that most of the DEGs associated with gained EPIs were significantly up-regulated with the average log2 fold-change > 0, while the DEGs corresponding to lost EPIs were down-regulated with the average log2 fold-change < 0 (Figure 5A), suggesting a positive correlation between changed EPIs and their corresponding DEGs. Enrichment analysis of GO functions and KEGG pathways revealed that these DEGs were significantly enriched (FDR <0.05) in regulations of chromatin organization, protein phosphorylation, RNA polymerase II transcription, and signaling pathways of breast cancer, EGFR and Notch (Figures 5B,C and Supplementary Table S5), such as up-regulated *EZH2*, *JRKL* and *KLF3* with gained EPIs and down-regulated *FOXA1*, *FA2H* and *MAPK13* with lost EPIs (Figures 5C–E, Supplementary Figure S6). The gain or loss of EPIs might be responsible for differential gene expression associated with doxorubicin resistance in breast cancer cells *via* enhancer reprogramming.

### *JRKL* and *FA2H* corresponded to changed EPIs and might be potential targets for acquired resistance to doxorubicin in breast cancer

**FIGURE 6:**
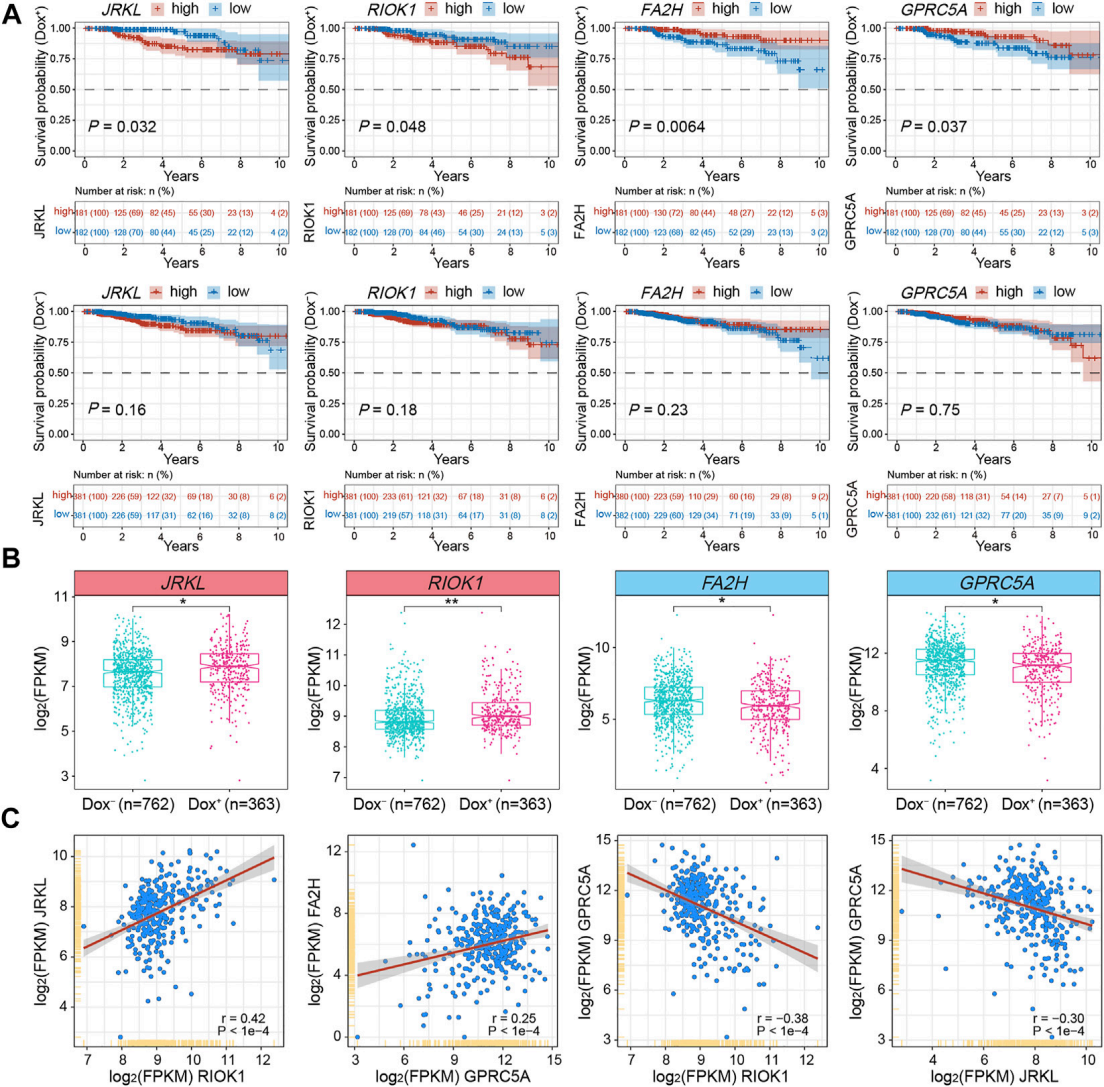
Integrative analysis of DEGs associated with changed EPIs using TCGA-BRCA clinical data and subtypes. **(A)** Kaplan-Meier survival analyses for *JRKL*, *RIOK1*, *FA2H* and *GPRC5A* of BRCA patients treated with (Dox^+^; top panel; *n* = 363) and without (Dox^−^; bottom panel; *n* = 762) doxorubicin (adriamycin). Expression values (FPKM) were sorted according to high and low expression from the median of the corresponding genes. The shaded area indicates the 95% confidence interval. Statistical analysis was performed by the log-rank test, and *p* < 0.05 was statistically significant. All the survival duration was censored to 10 years **(B)** Box plots showing the mRNA expression levels (FPKM) of *JRKL*, *RIOK1*, *FA2H* and *GPRC5A* in Dox^+^ and Dox^−^ breast cancer subgroups derived from TCGA datasets. The *p* values were calculated by Wilcoxon’s signed-rank test in the R platform. *p < 0.05, **p < 0.01. **(C)** Correlation analysis of mRNA expression levels (FPKM) among *JRKL*, *RIOK1*, *FA2H* and *GPRC5A* in the Dox^+^ breast cancer subgroup. The Pearson’s correlation coefficients (r) and *p* values were calculated using the “corrplot” package in R. The *p* values <0.05 were considered to be statistically significant.

To testify the expression patterns and the clinical significance of critical DEGs related to gained or lost EPIs in MCF7-DR cells as well as predict their role in the development of acquired resistance to doxorubicin in breast cancer, we retrieved the genomic and clinical data from TCGA (The Cancer Genome Atlas) BRCA (breast invasive carcinoma) and analyzed their expression levels in breast cancer patients treated with (Dox^+^; *n* = 363) and without (Dox^−^; *n* = 762) doxorubicin alone. Correlation analysis showed that the expression pattern of *JRKL* was positively correlated with *CCDC82* expression (r = 0.85), both of which were negatively correlated with *FOXA1*; the expression pattern of *E2F3* was positively correlated with *EZH2* (*r* = 0.53) and *RIOK1* (*r* = 0.53) expression (Figure 5F and Supplementary Table S6). Kaplan-Meier survival analysis further revealed that higher expression of *JRKL* and *RIOK1* was verified to be significantly (*p* < 0.05) associated with the poor prognosis of the Dox^+^ patients when compared to the Dox^−^ patients, whereas lower expression of *FA2H* and *GPRC5A* was related with the poor prognosis of Dox^+^ patients (Figure 6A). The mRNA expression levels of *JRKL* and *RIOK1* were significantly (*p* < 0.05) higher in the Dox^+^ patients than Dox^−^, while *FA2H* and *GPRC5A* expression levels were lower in the Dox^+^ patients (Figure 6B). Moreover, in the Dox^+^ patients, there was a positive correlation between the expression levels of *JRKL* and *RIOK1*, *FA2H* and *GPRC5A*, while *GPRC5A* expression was negatively correlated with *JRKL* and *RIOK1* as expected (Figure 6C, Supplementary Table S6). Therefore, the gained and lost EPIs and their corresponding DEGs, such as *JRKL*, *RIOK1*, *FA2H*, *GPRC5A* might play a critical role in the development of doxorubicin resistance in breast cancer cells and be important indicators for judging the prognosis or a therapeutic target in breast cancer patients treated with doxorubicin.

## Discussion

Despite an increasing number of molecular mechanisms underlying doxorubicin resistance in breast cancer have been reported (Huang et al., 2018; Stefanski et al., 2019; Mirzaei et al., 2021; Zangouei et al., 2021), it remains a major obstacle to improving the clinical benefit of cancer patients. Previous studies have shown that chromatin interactions in breast cancer cells differ from normal mammary epithelial cells (Barutcu et al., 2015) and estrogen-receptor binding is involved in three-dimensional (3D) genome reorganization in endocrine-resistant breast cancer (Zhou et al., 2019; Achinger-Kawecka et al., 2020). Here we generated high-resolution 3D genome maps using *in situ* Hi-C and performed integrated analyses of Hi-C, ATAC-seq and mRNA-seq experiments in doxorubicin-resistant MCF7 cells, in comparison to the parental MCF7 cells. Our multiscale analyses of 3D genome structure uncovered alterations in A/B compartments, topologically associating domains and chromatin loops. It was found that the genomic loci switch between A and B compartments, which are associated with active/open and inactive/closed chromatin respectively, was positively correlated with differential gene transcription. The DEGs located in compartments that switch from A-type to B-type in doxorubicin-resistant cells were mostly repressed, whereas DEGs that switched from A-type to B-type compartments showed significantly higher expression levels. The DEGs located in unchanged compartments showed a well-balanced expression level. These results are thus basically consistent with existing knowledge (Barutcu et al., 2015; Fan et al., 2018).

At the TAD and loop scale, we found that the genomes in MCF7-DR cells contain more TADs and the average TAD size is smaller than in MCF7 cells, indicating that there were more gained (newly formed) TAD boundaries and loop anchors in MCF7-DR cells. Combining ATAC-seq and mRNA-seq data, we further found that TAD boundaries and loop anchors possessed higher chromatin accessibility/openness, which would be in favor of the recruitment of TFs and insulator element-binding proteins, including insulator proteins CTCF and BORIS, which have been proposed to be indispensable for the formation of TADs and loops (Dixon et al., 2016; Debruyne et al., 2019; Hu et al., 2020; Ghosh and Meyer, 2021). Indeed our previous studies have found that BORIS was significantly up-regulated in MCF7-DR cells (Wang et al., 2021). Besides, some other TFs related to CTCF were also found to be enriched at TAD boundaries and loop anchors, such as HIC1 (Xu et al., 2021) and members of FOX TF family (Hurtado et al., 2011). Although the statistical significance of the enrichment of these TF binding motifs is obviously lower than that of CTCF and BORIS, they are worthy of further exploration in subsequent trials. In addition, both TAD boundaries and loop anchors show stronger insulations, suggesting genomic and functional overlap between them. In terms of gene transcription, the intensities of the mRNA product signals enriched at TAD boundaries were higher than those at loop anchors, indicating that TAD boundaries are enriched for active gene transcription (Rowley et al., 2017). Chromatin loops usually promote long-range interactions between transcriptional regulatory elements and their associated promoters (Rao et al., 2014; Mcarthur and Capra, 2021), and most of their anchors are located at the intergenic regions epigenomically defined as enhancers, which may be a reason for no significant enrichment of the mRNA-seq signals at loop anchors.

By comparing MCF7-DR cells with MCF7 cells, we observed that the gained TAD boundaries were enriched with higher ATAC-seq signals and vice visa, demonstrating a positive correlation between chromatin accessibility changes and TAD boundary strength. Besides CTCF and BORIS, members of AP-1 TF family are potentially involved in the formation of the gained TAD boundaries, while the lost TAD boundaries might be related to the weakened binding affinity of members of DLX and LHX TF families. Similarly, bHLH family members might contribute to gained chromatin loops associated with CTCF (Hu et al., 2020). In addition, interestingly, we found that there are two condensed chromatins (∼20 kb) with lower ATAC-seq signals flanking (within ∼30 kb) both conserved and changed (gained or lost) TAD boundaries, which may play critical roles in the formation and/or maintenance of TAD boundaries and have not been reported in any detail. In our opinion, the structure and function of these two special chromatin structures may be conserved and stable across cell types and species and deserve further study.

Alterations in the intrinsic chromatin interaction landscape are associated with differential gene expression (Wu et al., 2017; Misteli, 2020; Mcarthur and Capra, 2021), and we came to a similar conclusion. In particular, we identified several gained and lost enhancer-promoter interactions (EPIs) and their corresponding DEGs such as *FA2H, FOXA1*, *JRKL* and *EZH2*, many of which were involved in chromatin organization, Notch and breast cancer signaling pathways and have been known to be closely related to drug resistance or epigenetic regulation in human cancer (Herrero et al., 2008; Roe et al., 2017; Toh et al., 2017; Nussinov et al., 2021). Additional analysis of clinical and genomic data from TCGA-BRCA further revealed that some DEGs were highly correlated with each other and exhibited similar expression patterns in both the MCF7-DR cells and the Dox^+^ breast cancer patients as expected. The higher expression of *JRKL* and *RIOK1* in patients treated with doxorubicin alone experienced significantly worse clinical outcomes, while lower expression levels of *FA2H* and *GPRC5A* had opposite prognostic effects. Especially, previous studies found that *FA2H* (Fatty acid 2-hydroxylase) depletion could lead to decreased chemosensitivity to cisplatin *via* inhibition of *AMPK* and activation of mTOR/S6K1/Gli1 pathway in gastric cancer (Yao et al., 2019), and turn different cancer cells resistant to PM02734 (Herrero et al., 2008). Here we speculated that the gain or loss of enhancer-promoter interactions and their corresponding DEGs such as up-regulated *JRKL* and down-regulate *FA2H* may constitute potential diagnostic or therapeutic targets for reversing doxorubicin resistance in breast cancer.

## Conclusion

Our findings provide genome-wide evidence for the reorganization of 3D genome structure and accompanying chromatin accessibility and gene expression changes occurring in doxorubicin-resistant breast cancer cells. However, additional validation and further investigations will be required to establish the direct link between 3D genome reorganization observed in doxorubicin-resistant cell lines and specific molecular changes that can be used as therapeutic targets in breast cancer patients. Collectively, these results extend our understanding and suggest a key epigenomic mechanism of doxorubicin resistance and offer a resource for further studies to explore future therapeutic applications.

## Data Availability

The datasets presented in this study can be found in online repositories. The names of the repository/repositories and accession number(s) can be found below: sequencing data deposited in the NCBI GEO under the accession number GSE205874 and GSE174152 have now been released and publicly available (https://www.ncbi.nlm.nih.gov/geo/query/acc.cgi?acc=GSE205874, https://www.ncbi.nlm.nih.gov/geo/query/acc.cgi?acc=GSE174152).
