# Supplementary material for: Reorganization of 3D chromatin architecture in doxorubicin-resistant breast cancer cells
Source: Front Cell Dev Biol. 2022 Aug 5;10:974750. doi: 10.3389/fcell.2022.974750 (PMC9393755; doi:10.3389/fcell.2022.974750)
Supplement: Supplementary file 7 [file Datasheet1.docx]

Supplementary Material

## Supplementary Figures


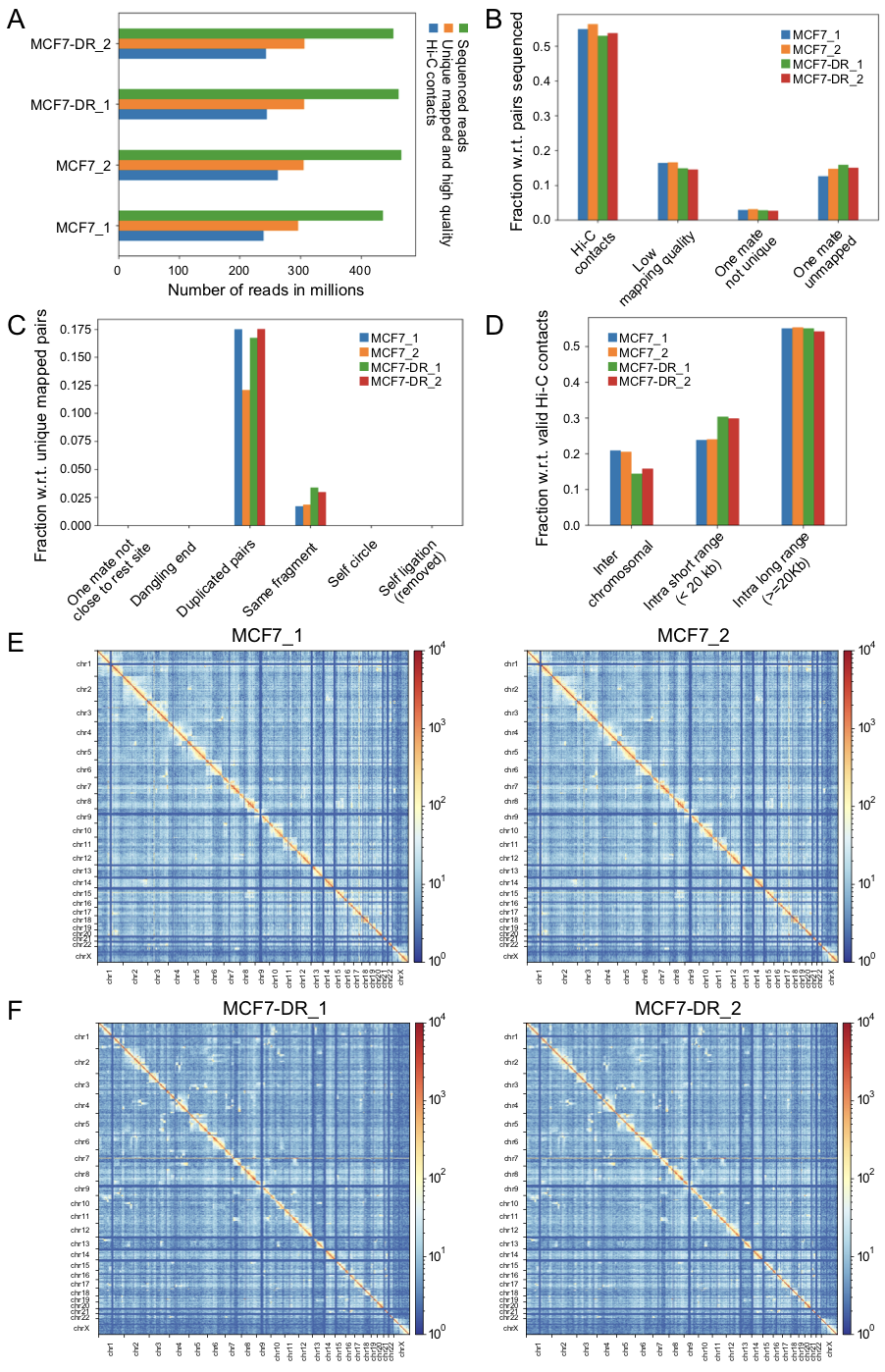


**Supplementary Figure S1. Generation of Hi-C interaction matrices and quality control.**

(A) Number of sequenced (green), unique mappable and high quality (orange), and valid used (blue) Hi-C reads per sequenced sample. The exact numbers of sequenced and valid reads are available in Table S1. Two independent biological replicates were sequenced for parental (MCF7_1 and MCF7_2) and doxorubicin-resistant (MCF7-DR_1 and MCF7-DR_2) breast cancer cells.

(B) Fraction of used, unmappable and non-unique read pairs with respect to the sequenced read pairs.

(C) Fraction of read pairs that were discarded when building the Hi-C matrix.

(D) Fraction of inter-chromosomal, short-range (< 20 kb) and long-range (> 20 kb) Hi-C contacts with respect to the valid read pairs used in this study.

(E and F) Heatmaps showing whole-genome Hi-C interaction matrix normalized at 1-Mb resolution. For each condition, two biological replicates in independent experiments were analyzed for parental (E) and doxorubicin-resistant (F) MCF7 cells.


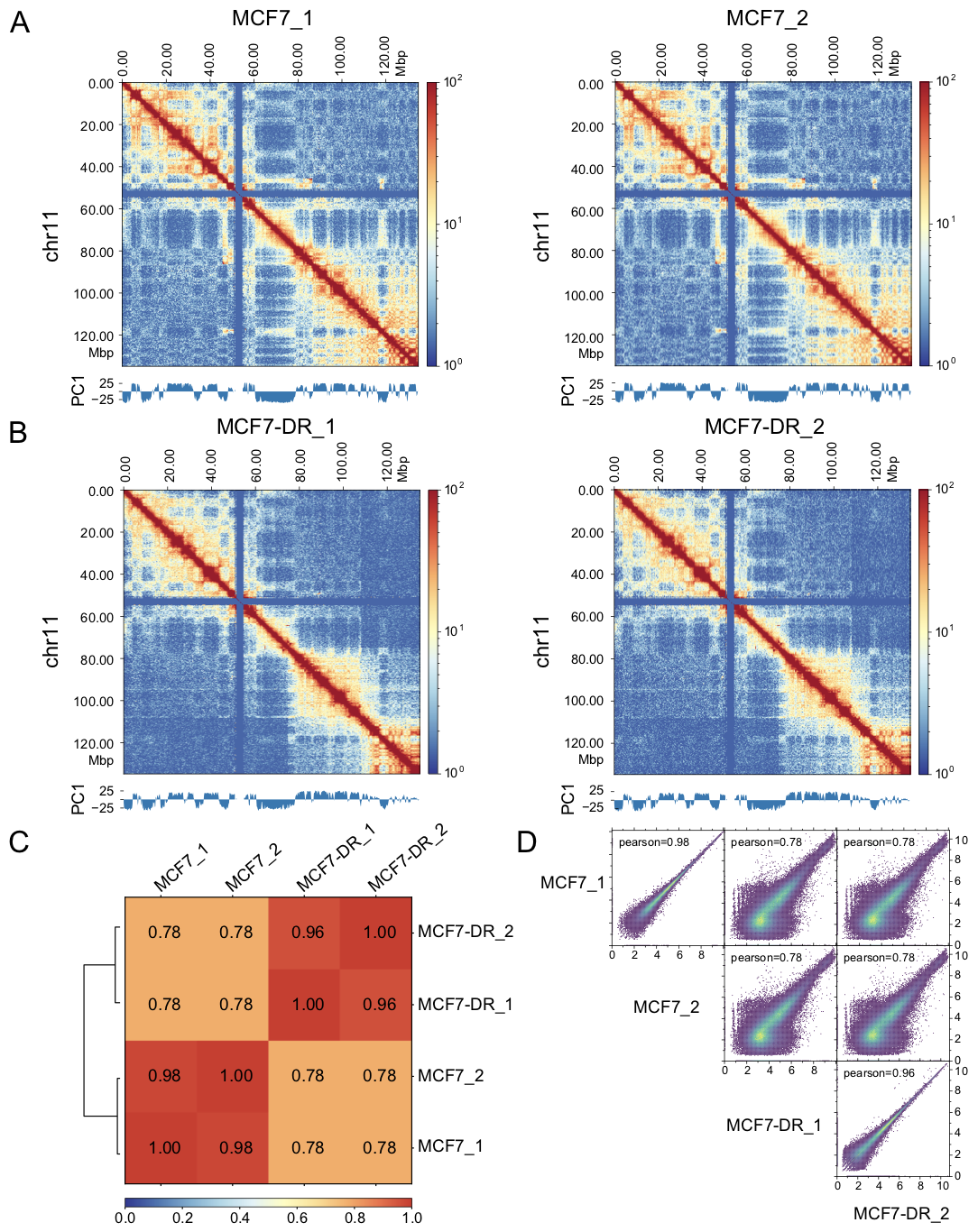


**Supplementary Figure S2. Example heatmaps and reproducibility of Hi-C replicates.**

(A and B) Images of whole-chromosome Hi-C interaction matrices of chr11 at 200 kb resolution. Two independent biological replicates were analyzed for MCF7 (A) and MCF7-DR (B) breast cancer cells. The PC1 signal was used to define the A and B compartments and is displayed below each heatmap (positive PC1 is designated as A compartments, and negative PC1 is designated as B compartments).

(C and D) Heatmap (C) and scatter plot (D) showing the Pearson correlation coefficient (PCC) of genome-wide interactions at 1-Mb resolution of all samples.


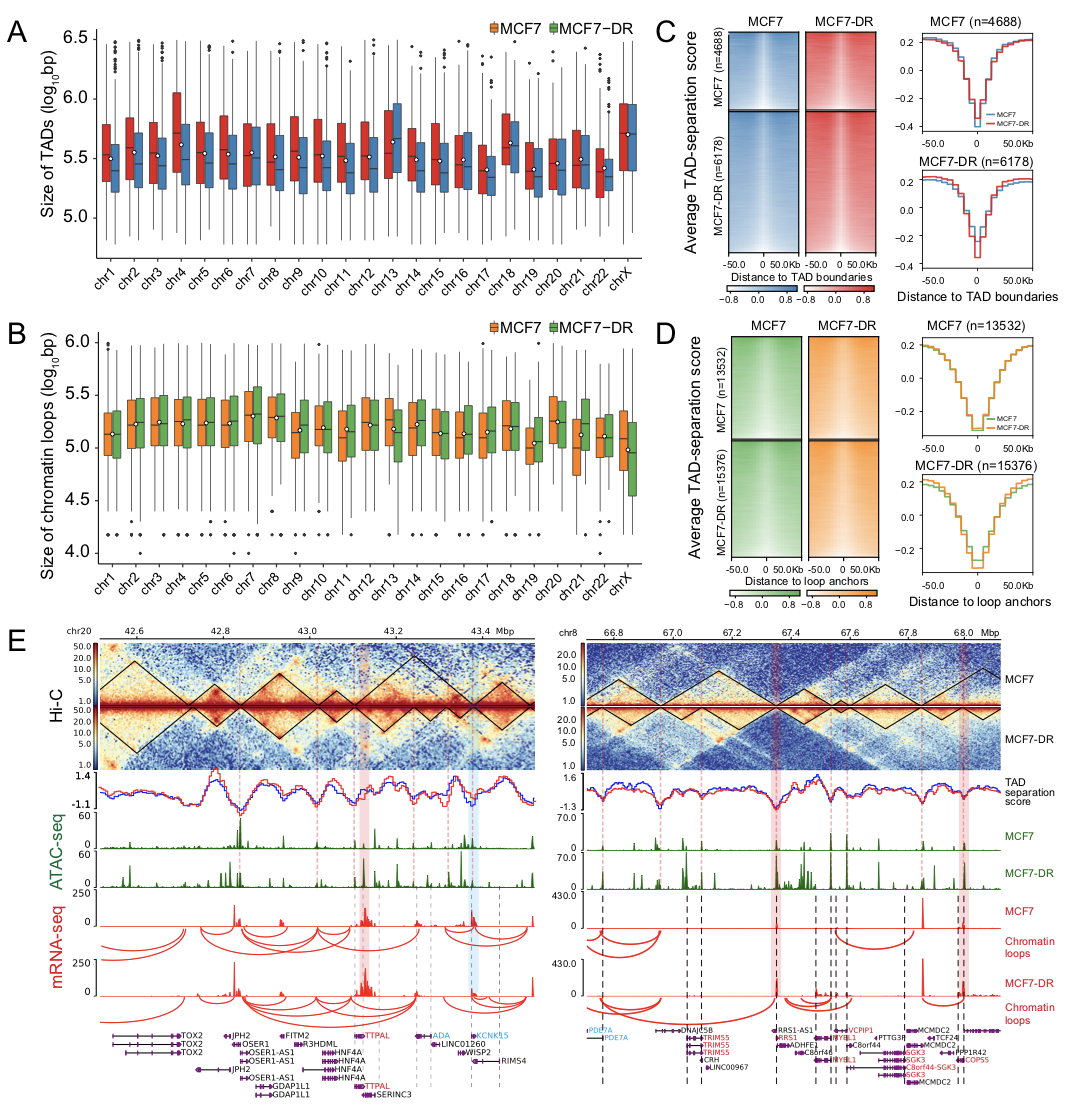


**Supplementary Figure S3. Identification and visualization of TADs and chromatin loops.**

(A and B) Box plots showing the size distribution of TADs (A) and loops (B) at 5-kb resolution for individual chromosome.

(C) Heatmaps and average profiles displaying average TAD-separation scores across a genomic window of -50 kb upstream and +50 kb downstream from the center of TAD boundaries at 5-kb resolution in parental and doxorubicin-resistant MCF7 cells. The top panels show read signals over the 4688 TAD boundaries identified in MCF7 cells and the bottom panels show read signals over the 6178 TAD boundaries identified in MCF7-DR cells.

(D) Heatmaps and average profiles showing average TAD-separation scores located within 50 kb upstream and downstream of the center of loop anchors at 5-kb resolution in MCF7 and MCF7-DR cells. The top panels show read signals over the 13532 loop anchors identified in MCF7 cells and the bottom panels show read signals over the 15376 loop anchors identified in MCF7-DR cells.

(E) Representative snapshots of Hi‐C interaction matrix at 5-kb resolution (top panel), and black line in the heatmap demarcates the identified TADs. TAD-separation scores of MCF7 (solid blue line) and MCF7-DR (solid red line) cells were calculated to identify the degree of separation between the left and right regions at each Hi-C matrix bin. The following tracks showing normalized ATAC-seq (green) and mRNA-seq (red) read coverage. The red shaded area shows hyper-accessible chromatin region and up-regulated genes, and the blue shaded area shows hypo-accessible chromatin region and down-regulated genes. Chromatin loops are displayed as red arcs under the mRNA-seq track. Hi-C contact matrix, TADs and chromatin loops were generated using HiCexplorer. The red vertical dashed lines indicate ATAC-seq signals at the TAD boundaries. The transcription start site (TSS) and transcription end site (TES) of DEGs are indicated by the black vertical dashed lines. Up-regulated genes are colored in red and down-regulated genes in blue, while unchanged genes are shown in black.


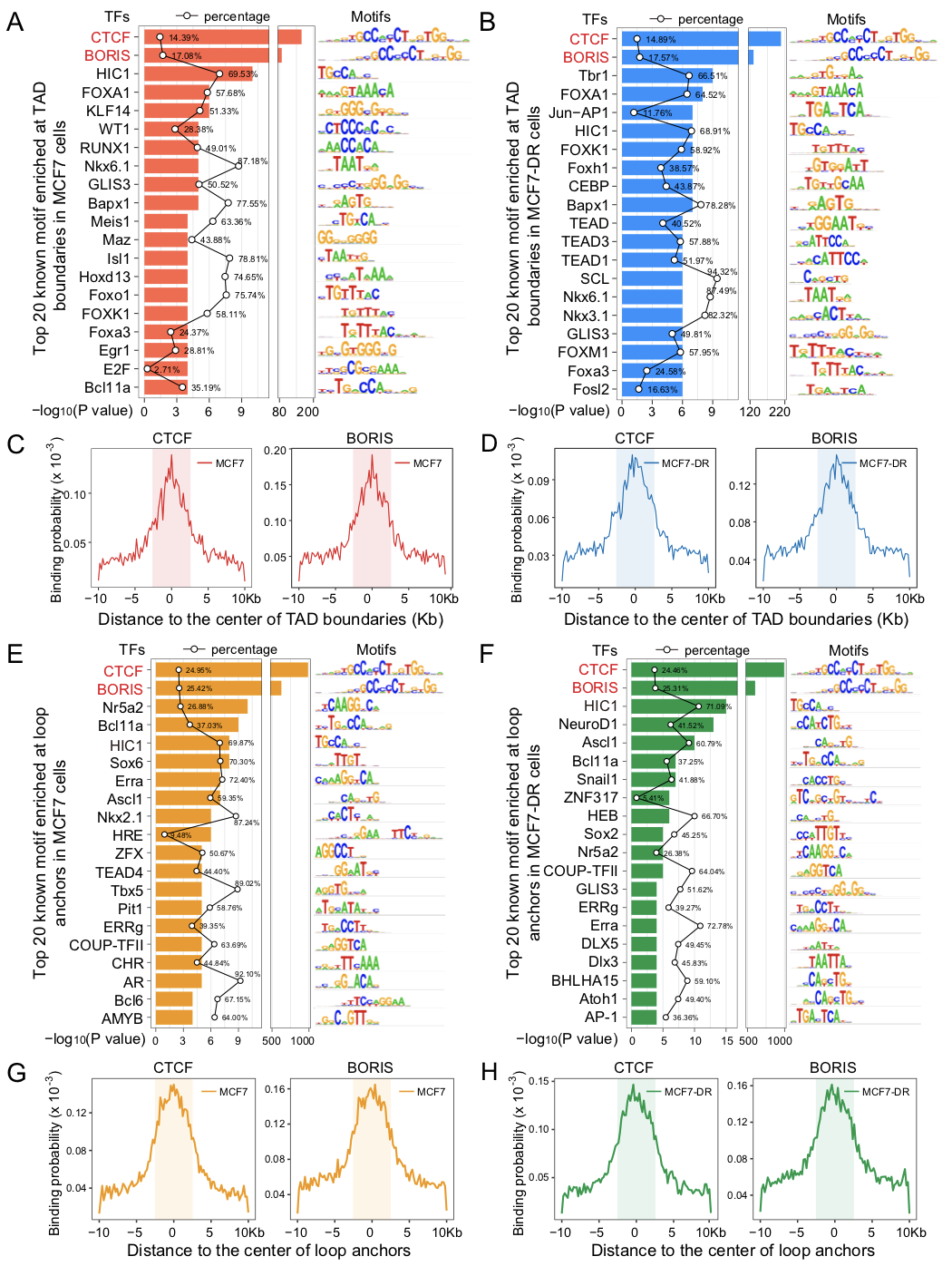


**Supplementary Figure S4. Transcription factors potentially enriched at TAD boundaries and loop anchors.**

(A and B) Top 20 known TF motifs enriched in TAD boundaries of MCF7 (A) and MCF7-DR (B) cells at 5-kb resolution, with P values estimated from HOMER v.4.10 using a binomial algorithm. The polygonal chain in black shows the percentages of target sequences with TF motifs.

(C and D) The aggregate profiles show the binding probability distribution of CTCF and BORIS motifs within 10 kb around the center of TAD boundaries at 5-kb resolution in MCF7 (C) and MCF7-DR (D) cells. The shaded areas represent the regions of TAD boundaries.

(E and F) Top 20 known TF motifs enriched in chromatin loop anchors of MCF7 (E) and MCF7-DR (F) cells at 5-kb resolution. The percentages of target sequences with TF motifs are indicated by the black polygonal chain. The P values for motif enrichment were performed by the HOMER v.4.10 software.

(G and H) The aggregate profiles representing the binding probability distribution of CTCF and BORIS motifs within 10 kb around the center of loop anchors at 5-kb resolution in MCF7 (G) and MCF7-DR (H) cells. The shaded areas indicate the regions of chromatin loop anchors.


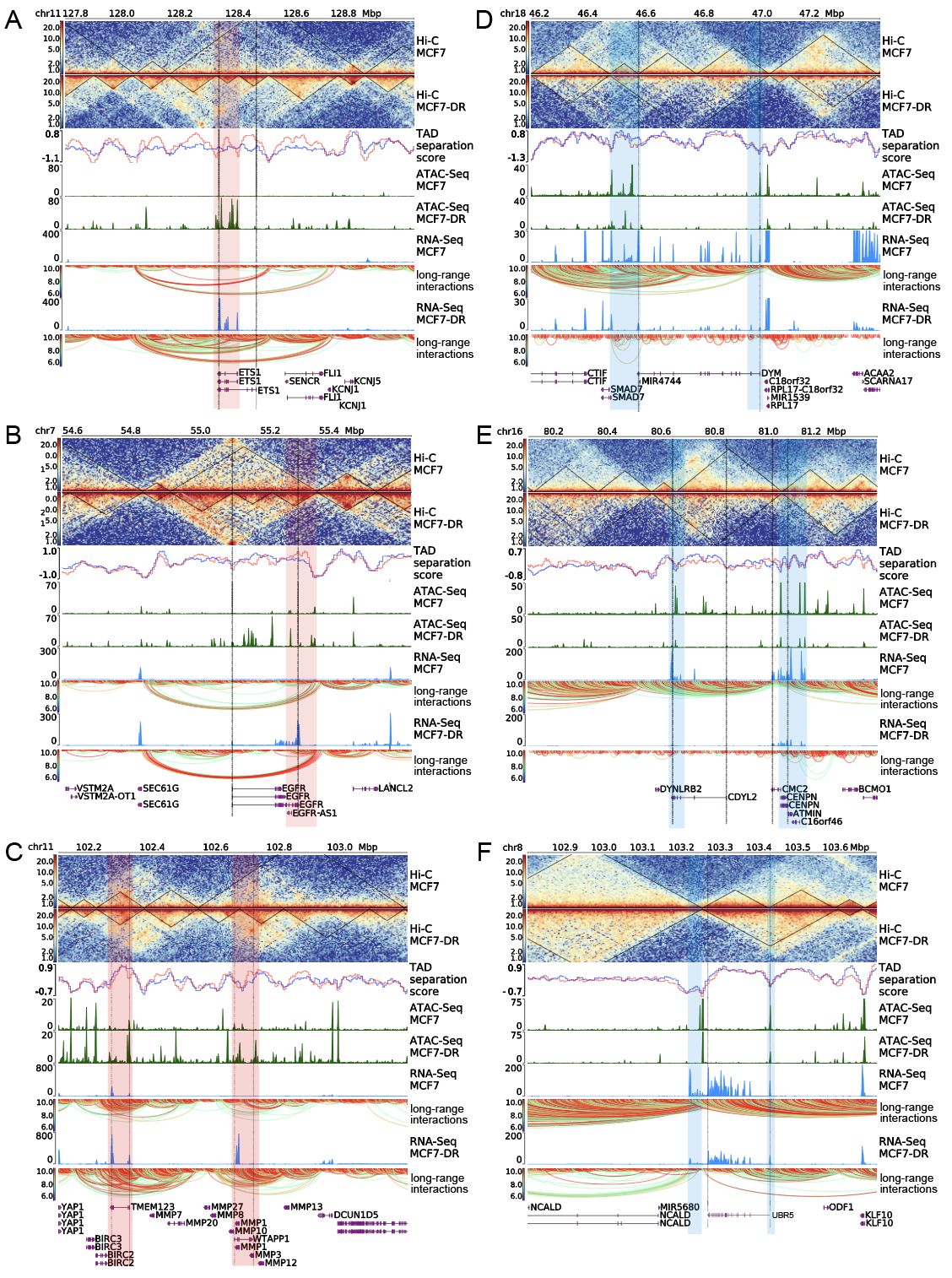


**Supplementary Figure S5. Representative snapshots of DEGs associated with long-range interaction changes.**

(A-F) Example regions showing Hi‐C interaction matrix at 5-kb resolution with TADs (delineated by solid black lines), TAD-separation score, compartment (PC1), ATAC-seq, mRNA-seq, and significant chromatin interactions. TAD-separation scores of MCF7 and MCF7-DR cells are shown by solid lines in blue and red, respectively. Red shaded areas indicate hyper-accessible chromatin regions and up-regulated genes (A-C), and blue shaded areas show hypo-accessible chromatin regions and down-regulated genes (D-F). Significant chromatin interactions (P < 0.05) identified by HOMER v4.10 using the analyzeHiC are represented by a rainbow scale ranging from 6 to 10. The black vertical dashed lines indicate the TSS or TES of DEGs.


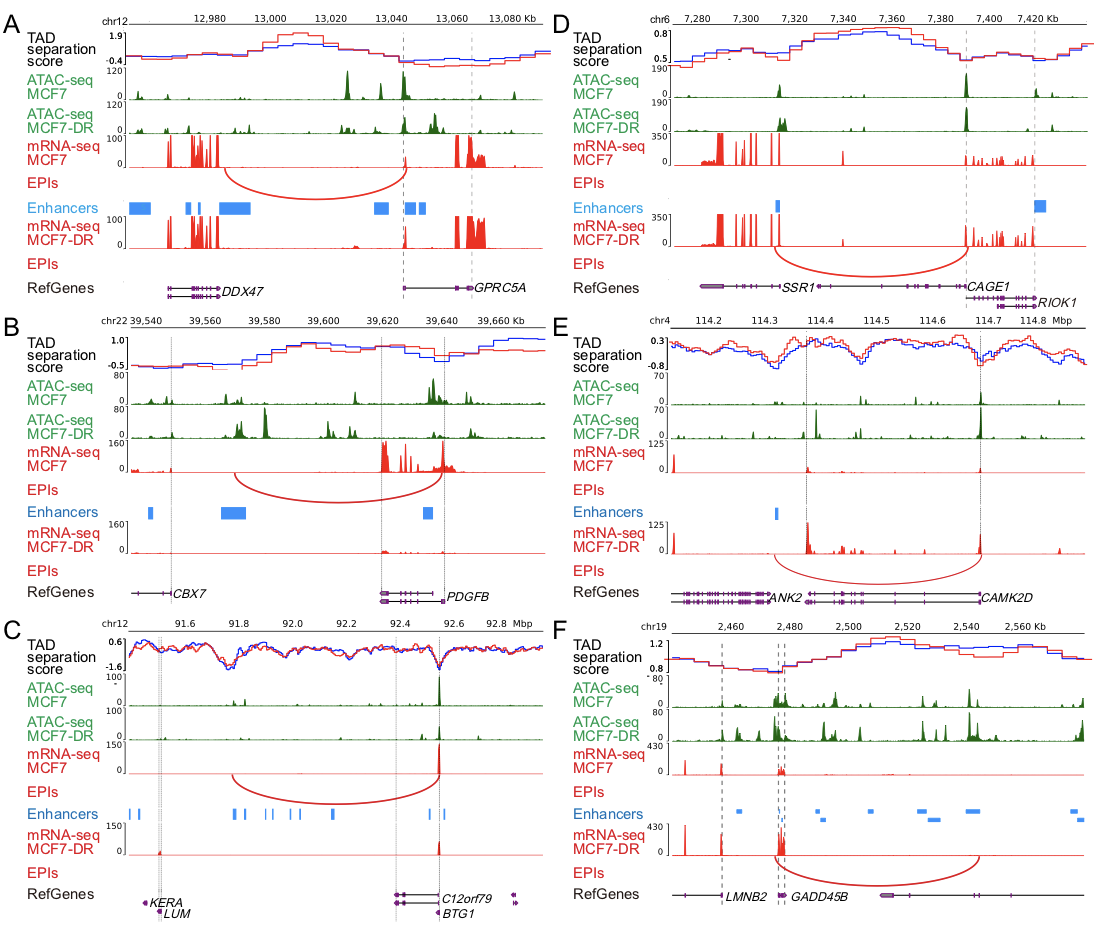


**Supplementary Figure S6. Representative DEGs corresponded to changed EPIs.**

(A-F) Representative snapshots of TAD-separation scores, ATAC-seq (green) and mRNA-seq signals (red), enhancers (blue), and lost (A-C) or gained (D-F) EPIs (red) of down-regulated (A-C) or up-regulated (D-F) DEGs in MCF7-DR cells compared to MCF7 cells. TAD-separation scores of MCF7 and MCF7-DR cells are shown as solid blue and red lines, respectively. The black vertical dashed lines indicate the TSS or TES of DEGs.

## Supplementary Tables

**Supplementary Table S1. Hi-C library sequencing statistics.**

**Supplementary Table S2. Identified topologically associating domains.**

**Supplementary Table S3. Identified chromatin loops.**

**Supplementary Table S4. Differential enhancer-promoter interactions (EPIs) and related DEGs.**

**Supplementary Table S5. GO and KEGG enrichment of DEGs associated with differential EPIs.**

**Supplementary Table S6. Pearson's correlation coefficient of identified DEGs associated with differential EPIs using TCGA-BRCA dataset (Dox^+^).**
